# Elevated Cardiac Troponin I in Sepsis and Septic Shock: No Evidence for Thrombus Associated Myocardial Necrosis

**DOI:** 10.1371/journal.pone.0009017

**Published:** 2010-02-03

**Authors:** David R. Altmann, Wolfgang Korte, Micha T. Maeder, Thomas Fehr, Philipp Haager, Hans Rickli, Gian-Reto Kleger, Regulo Rodriguez, Peter Ammann

**Affiliations:** 1 Division of Cardiology, Kantonsspital St. Gallen, St. Gallen, Switzerland; 2 Institute for Clinical Chemistry and Hematology, Kantonsspital St. Gallen, St. Gallen, Switzerland; 3 Baker IDI Heart and Diabetes Institute, Melbourne, Australia; 4 Division of Nephrology, University Hospital Zürich, Zürich, Switzerland; 5 Intensive Care Unit, Department of Internal Medicine, Kantonsspital St. Gallen, St. Gallen, Switzerland; 6 Institute of Pathology, Kantonsspital St. Gallen, St. Gallen, Switzerland; Lerner Research Institute, United States of America

## Abstract

**Background:**

Elevated cardiac troponin I (cTnI) is frequently observed in patients with severe sepsis and septic shock. However, the mechanisms underlying cTnI release in these patients are still unknown. To date no data regarding coagulation disturbances as a possible mechanism for cTnI release during sepsis are available.

**Methodology/Principal Findings:**

Consecutive patients with systemic inflammatory response syndrome (SIRS), sepsis or septic shock without evidence of an acute coronary syndrome were analyzed. Coagulation parameters (clotting time (CT), clot formation time (CFT), maximum clot firmness (MCF), α-angle) were assessed in native whole blood samples, and using specific activators to evaluate the extrinsic and intrinsic as well as the fibrin component of the coagulation pathway with the use of rotational thrombelastometry (ROTEM).

Thirty-eight patients were included and 22 (58%) were cTnI-positive. Baseline characteristics between TnI-positive and -negative patients were similar. The CT, CFT, MCF and the α-angle were similar between the groups with trends towards shorter CT in the extrinsic and fibrin activation.

**Conclusions/Significance:**

We found no differences in coagulation parameters analyzed with rotational thrombelastometry between cTnI-positive and -negative patients with SIRS, severe sepsis, and septic shock. These findings suggest that pathophysiological mechanisms other than thrombus-associated myocardial damage might play a major role, including reversible myocardial membrane leakage and/or cytokine mediated apoptosis in these patients.

## Introduction

Elevated concentrations of cardiac troponin I (cTnI) are frequently observed in patients with severe sepsis and septic shock even in the absence of an acute coronary syndrome (ACS) [1]. Elevated cardiac cTnI in this setting are associated with left ventricular dysfunction [2] and adverse outcome [3]. The mechanisms underlying cTnI release in patients with sepsis are still unknown, and a number of hypotheses have been formulated [4]. Pre-existing coronary artery disease (CAD) and demand ischemia could account for cTnI release in sepsis. However, we have shown that cTnI release can also occur in patients in whom significant CAD has been excluded with sensitive methods [5]. The coagulation system is activated in critically ill patients and disseminated intravascular coagulation frequently occurs. A procoagulant state resulting in microvascular thrombi may promote organ dysfunction in sepsis. There is ample evidence from the literature that platelet properties such as reactivity and receptor density are associated with an increased risk of troponin release with reduced coronary perfusion [6]–[8]. Thus, one might speculate that septic patients with increased troponin concentrations might show evidence of increased platelet reactivity; in fact, it has been shown that septic patients show increased platelet-leukocyte interaction [9], which is found early in myocardial infarction [10] and which is associated with an increased troponin release in patients undergoing percutaneous coronary intervention; interestingly, this increase can be inhibited with the use of a GP IIb/IIIa inhibitor [11]. The aim of the present study was to compare properties of ex vivo clot formation between cTnI-positive and -negative patients with systemic inflammatory response syndrome (SIRS) and sepsis. Therefore, we measured properties of clot formation in SIRS and septic patients with the help of thrombelastometry.

## Methods

### Ethic Statement

All patients, or in case of unconsciousness, their closest relatives, signed a written informed consent. The study was approved by the ethics comittee of the Kanton St. Gallen, Switzerland.

### Patients

Thirty-eight consecutive patients referred to our medical intensive care unit and fulfilling criteria for severe sepsis, septic shock or SIRS were studied. Definitions for sepsis, septic shock, and SIRS correspond to the criteria of the consensus conference of the American College of Chest Physician and Society of Critical Care Medicine [12]. Patients with evidence of an ACS defined as chest pain with either ST-elevation, depression (>1 mm) or T-wave inversions were excluded, as well as patients with a history of CAD or with a recent (<14 days) history of cardiac surgery. In unconscious patients ST-T abnormalities as described above were sufficient to assume an ACS.

### Study Course

A 12-lead ECG was obtained at study inclusion and after 12 and 24 hours. The simplified Acute Physiology Score II (SAPS-II) was used to describe/assess disease severity at study entry [13]. An experienced cardiologist blinded to cTnI levels performed transthoracic echocardiography to assess left ventricular ejection fraction (LVEF), using the biplane Simpson formula [14] within 24 hours of study inclusion, and in case of hemodynamic deterioration during the study period. Standard therapy such as antimicrobial therapy, renal replacement therapy, mechanical ventilation or hemodynamic support, was not influenced by the study and was at the physicians' discretion. Survival was assessed 28 days after study inclusion. In case of death, an autopsy was performed when ever possible. To exclude flow-limiting CAD in cTnI-positive survivors, dobutamine stress echocardiography was performed according to standard procedures [15] within 4 months after recovery.

### Laboratory Analyses

**Figure 1:**
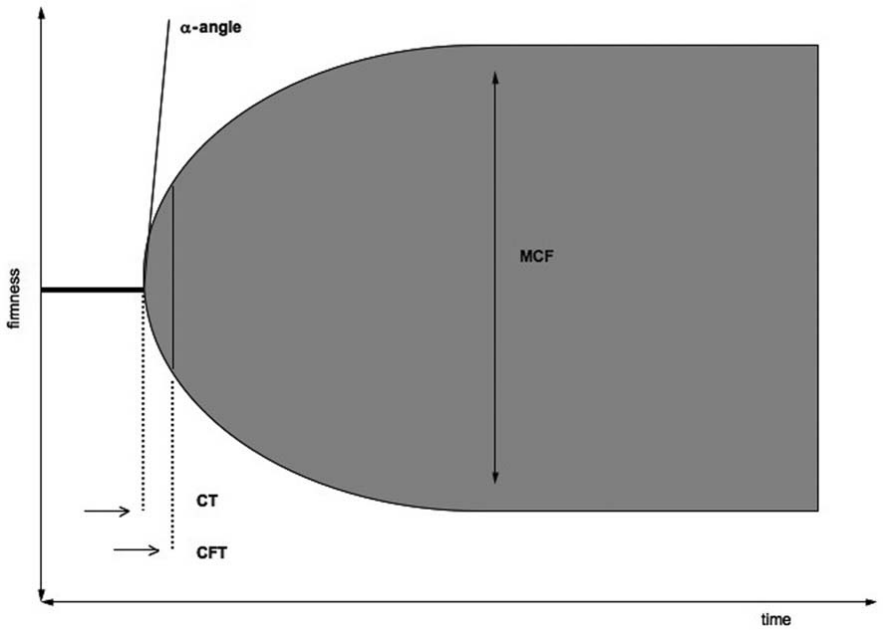
Example of the rotational thrombelastometry analysis. Liquid whole blood transmits no amplitude on the rotational thrombelastograph tracing. As blood clots start to form, fibers composed of fibrin and platelets produce increasing amplitude in the rotational thrombelastograph tracing. Hypercoagulable blood would typically show a shorter CT and CFT with a higher MCF and a steeper α-angle. CT = clotting time: time between starting the reaction and first detection of fibrin generation → thrombin formation, start of fibrin polymerisation; CFT = clot formation: time from initiation of clotting until a defined clot strength can be first detected → fibrin polymerization, clot stabilization with thrombocytes and F XIII; MCF = maximal firmness of the clot: maximum amplitude recorded, firmness of the clot → increasing clot stabilisation by polymerized fibrin, thrombocytes and F XIII.

Blood was obtained from a radial arterial line and cTnI was measured at the time of study inclusion and after 12, 24, 48, 96, 192 and 288 hours and analyzed from the serum on a day by day basis with a second-generation assay (Access AccuTnI Troponin I; Beckman Coulter Inc; Fullerton, California). Two consecutive cTnI values >0.5 µg/l were considered “cTnI-positive”. In addition, creatine phosphokinase (CK), CK-MB activity and C-reactive protein (CRP) were assessed. Blood samples for hemostasis analysis were taken at study inclusion, and after 12, 24 and 48 hours and analyzed using rotational thrombelastometry (ROTEM®; Pentapharm GmbH; Munich, Germany). The method and the parameters of thrombelastometry have been described in detail previously [16]. Briefly, thrombelastometry evaluates the clotting process in vitro by assessment of the viscoelastic properties in whole blood that occur during clot activation, clot formation and stabilization as well as clot lysis (figure 1). Thrombelastometry is a reliable method to evaluate perioperative hypercoagulability [17] and has been widely used to monitor coagulation and specifically guide blood component therapy in trauma patients [18], abdominal surgery [19] and more widely in hepatic [20] and cardiac surgery [21], conditions where coagulation disorders are frequent. For clarity the following parameters measured at maximum cTnI-level are explained: the clotting time (CT), the clot formation time (CFT), the maximum clot firmness (MCF) and the α-angle. The CT reflects the time between starting the reaction and first detection of fibrin generation. The CFT reflects the time until defined clot strength is reached, representing clot generation by plasmatic and cellular (platelets) components. The MCF corresponds to the maximum amplitude recorded by the thrombelastograph, which is equivalent to the maximum tensile strength of the whole clot. The α-angle assesses the velocity of fibrin polymerization, thus the speed of clot formation. Hypercoagulable blood would typically show a shorter CT and CFT with a higher MCF and steeper α-angle.

All coagulation parameters were measured in whole blood samples without addition of specific reagents (NATEM), and using specific activators to evaluate the extrinsic (EXTEM) and intrinsic (INTEM) pathway of the coagulation system. In addition, by blocking platelets the fibrin component of the coagulation (FIBTEM) was analyzed separately. Thus, through analysis of whole blood samples, thrombelastometry assesses cellular and plasmatic components simultaneously, allowing the plasmatic coagulation system to interact with cellular components, and therefore provides complete information during the process of clot initiation, formation and stabilization [16].

### Statistical Analysis

Categorical data are presented as numbers (percentages) and continuous data as mean ± standard deviation or median values (interquartile ranges, IQR) as appropriate. Fisher's exact test was used to compare categorical variables, and Mann Whitney-U tests were used to compare continuous data. A p-value <0.05 was considered statistically significant. Statistical calculations were performed using the statistical package SPSS 16 for Mac (SPSS Inc. USA).

## Results

Demographic data of the thirty-eight included patients are presented in table 1. Six patients (16%) were aplastic due to chemotherapy for hematologic malignancies. The rate of intravenous heparin treatment was similar in both groups and no patient received other anticoagulant therapy or platelet transfusion during the study period. The majority had severe sepsis (19%) or septic shock (42%). None of the study patients had symptoms or clinical signs of manifest CAD at study inclusion. However, non-specific ST-segment alterations (<1 mm) were found in three (13%) cTnI-positive, and in one (6%) cTnI-negative patient.

**Table 1:** Baseline characteristics of the study population (n = 38).

Age (yrs)	66±14
Male gender	19 (50%)
Body Mass Index (kg/m^2^)	27±5
SAPS-II (score points)	55±23
Systemic inflammatory response syndrome	10 (26%)
Sepsis	5 (13%)
Severe sepsis	7 (19%)
Septic shock	16 (42%)
Main diagnosis	
Pneumonia	14 (37%)
Cholezystitis/cholangitis	3 (8%)
Erysipelas	3 (8%)
Colitis	2 (5%)
Pancreatitis	1 (3%)
Peritonitis	2 (5%)
Meningitis	1 (3%)
Urosepsis	1 (3%)
Type of pathogen	
Gram positive	15 (40%)
Gram negative	11 (29%)
Fungal infection	2 (5%)
Culture negative	10 (26%)

Data are presented as numbers (%) and mean ± standard deviation as appropriate. SAPS-II = simplified acute physiology score II (high scores indicate severe illness).

Median values of LVEF, laboratory parameters such as CRP, creatinine, and CK, supportive therapy by means of mechanical ventilation, renal replacement therapy, and the use of catecholamines did not differ between cTnI-positive and -negative patients. In addition, disease severity indicated by SAPS-II at study inclusion was similar in cTnI-positive and -negative patients (table 2).

**Table 2:** Baseline characteristics other than coagulation between cTnT+ and cTnT− patients with SIRS or sepsis.

Covariates	cTnI+ (n = 22)	cTnI− (n = 16)	p value
Age (yrs)	65±13	63±13	0.77
Male gender	10 (46%)	9 (56%)	0.74
Body Mass Index (kg/m^2^)	29±7	27±6	0.39
Aplastic	2 (9%)	4 (25%)	0.18
Systolic blood pressure (mmHg)	104±24	111±17	0.31
Diastolic blood pressure (mmHg)	61±15	59±7	0.65
Heart rate (bpm)	102±19	98±17	0.58
SAPS-II (points)	55±22	54±26	0.89
Septic shock	8 (37%)	8 (50%)	0.50
Left ventricular ejection fraction (%)	58±14	58±12	0.98
Mechanical ventilation	14 (64%)	8 (50%)	0.51
Renal replacement therapy	10 (45%)	6 (38%)	0.74
C-reactive protein (mg/l, <8.0)	334±121	357±135	0.57
Creatinine (µmol/l, m<115; f<95)	141 (101–245)	119 (96–160)	0.27
Creatinephosphokinase (U/l, <170)	224 (78–1650)	151 (30–529)	0.16
Hemoglobin (g/l)	96±20	93±12	0.60
Troponin I (mg/l)	1.7 (0.8–4.6)	0.1 (0.07–0.3)	0.0001
Mortality	6 (27%)	4 (25%)	0.99

Data are presented as numbers (%) and mean ± standard deviation or median (IQR) as appropriate. SAPS-II = simplified acute physiology score II (high scores indicate severe illness).

### Clotting Parameters

No differences in the coagulation parameters CT, CFT, MCF and the α-angle analyzed with the use of rotational thrombelastometry were found between cTnI-positive and -negative patients. In EXTEM and FIBTEM trends towards shorter CT were found (table 3).

**Table 3:** Coagulation parameters.

	cTnI+	cTnI−	p value
**NATEM**			
CT	672 (564–990)	764 (602–1104)	0.50
CFT	182 (112–239)	138 (64–247)	0.44
MCF	57 (38–67)	57 (38–64)	0.74
α-angle	62 (43–70)	52 (41–66)	0.59
**INTEM**			
CT	188 (177–217)	200 (192–228)	0.25
CFT	66 (50–143)	56 (50–78)	0.39
MCF	61 (46–70)	56 (47–69)	0.81
α-angle	76 (72–79)	78 (75–79)	0.62
**EXTEM**			
CT	64 (55–71)	70 (62–79)	0.08
CFT	74 (57–158)	78 (61–101)	0.90
MCF	62 (51–69)	58 (46–70)	0.72
α-angle	75 (71–79)	76 (72–78)	0.62
**FIBTEM**			
CT	64 (57–68)	70 (60–77)	0.09
CFT	90 (61–201)	87 (68–141)	0.53
MCF	30 (23–35)	34 (27–40)	0.13
α-angle	75 (69–77)	75 (72–76)	0.85

Data are presented as median (IQR). NATEM = native coagulation (without addition of specific reagents); INTEM, EXTEM = intrinsic and extrinsic coagulation pathway (specific activators are added); FIBTEM = fibrin component of the coagulation (platelets are specifically blocked). See figure 1 for abbreviations of the clotting parameters.

### Troponin Status and Flow Limiting CAD

Twenty-two (58%) of all study patients were cTnI-positive. Median cTnI level in cTnI-positive patients was 1.7 µg/l (0.8–4.6). Among the sixteen cTnI-positive survivors, significant flow limiting CAD could be excluded by dobutamine stress-echocardiography after recovery from disease in seven patients (44%), and by coronary angiography in one patient (6%). In the remaining eight cTnI-positive survivors, one had a normal coronary angiogram performed one day before study inclusion, and in two patients a recent (<6 months) non-invasive cardiac exercise testing (myocardial perfusion scintigraphy in one patient and cycle ergometer testing in another patient) did not reveal significant CAD. Thus, in 69% (11/16) of cTnI-positive survivors, significant CAD could be ruled out. Ten patients of the entire study population (26%), 6/22 (27%) cTnI-positive and 4/16 (25%) cTnI-negative patients had died. Autopsy was performed in seven patients; thereof four patients (57%) were cTnI-positive. Of those three had normal coronary arteries, and one patient had CAD with a thrombotic obstruction of the left anterior descending artery and cardiomyocyte necrosis representing an acute myocardial infarction. In three cTnI-negative patients, no flow-limiting CAD was found at autopsy. Overall we could exclude relevant CAD in 64% (14/22) of cTnI-positive patients. Mortality assessed 28 days after study inclusion did not differ between cTnI-positive and -negative patients.

## Discussion

We hypothesized that cTnI elevation in SIRS and septic patients might be a consequence of microvascular flow disturbance provoked by a hypercoagulable state resulting in cardiomyocyte damage or death. Therefore, we prospectively investigated clotting parameters in 38 patients with SIRS and sepsis with the use of rotational thrombelastometry. This is to our knowledge the first study investigating the coagulation system in SIRS and septic patients with respect to their cTnI-status. However, no differences in clotting parameters as assessed by rotational thrombelastometry between cTnI-positive and -negative patients were found. Furthermore we ruled out relevant CAD as an alternative cause for cTnI elevation in the majority of the cTnI-positive patients.

It has been shown that thrombelastographic variables reflect additional information on the hemostatic process compared to common coagulation laboratory parameters, such as fibrinogen or activated partial thromboplastin time [22]. A recently published study used thrombelastometry, plasmatic coagulation parameters and platelet count to assess the coagulation system in septic patients before and during a short-term antithrombin therapy. The authors describe a hypercoagulable state in all septic patients before treatment as assessed by thrombelastometry [23]. As the coagulation status assessed with thrombelastometry is performed in whole blood, this technique seems to mirror the overall coagulation effects more closely than classical clotting parameters or activation markers (e.g. thrombin-antithrombin complexes) as all components of the blood - plasmatic, cellular components and platelets - can contribute to clot formation. Thrombelastometry detects a hypercoagulable state in patients who have experienced a venous or arterial thrombotic event [24]. However, it has to be kept in mind that thrombelastometry is relatively insensitive to changes in the so-called “primary hemostasis” (e.g. platelet dysfunction, von Willebrand disease etc.).

How can our findings be interpreted in context to previous studies? Several decades ago Parillo et al. described a circulating myocardial depressant substance in septic patients who showed reversible depression of LVEF [25]. Later, it turned out that Interleukin-6 (IL-6) and Interleukin-1β (IL-1β) play a major role in left ventricular dysfunction in septic patients [26]. However, the observation that severely impaired LVEF in septic patients most often improves after disease recovery speaks against a major myocardial cell death in septic patients. Interestingly, cytokine mediated leakage of structural proteins such as troponins could be demonstrated in vitro [27].

This hypothesis is supported by our previous clinical study in septic patients showing significantly elevated tumor-necrosis-factor α (TNFα) and IL-6 in troponin-positive as compared to -negative patients [5]. However, thrombus-mediated microvessel occlusion, as an underlying cause for troponin elevation in septic patients has never been studied as a possible mechanism.

### Limitations

The complexity of the human coagulation system is difficult to assess by a single laboratory analysis, and other changes of the coagulation system not detected by thrombelastometry might play a role in this setting. As this is an exploratory study we cannot exclude that other components of the coagulation system (that were not evaluated in the current study) might be important with regard to troponin positivity. Classical thrombelastography is rather insensitive to platelet function. Despite the increased robustness of the ROTEM assay, we cannot exclude that ROTEM analysis assessed from arterial blood specimens is not sensitive enough to detect minor coagulation differences in cardiac microcirculation between troponin positive and negative patients.

The small sample size is a limitation to our study. We did not explore the impact of disease severity (e.g. from SIRS to septic shock) on the coagulation system between cTnI-positive and –negative patients due to the limited sample size. Given the trends towards shorter CT times in EXTEM and FIBTEM, one could speculate that the results may have been different had more patients been included. However, we are aware of the difficulties to assess the function of the coagulation system “ex vivo”.

### Conclusion

In a relatively small group of patients with SIRS, sepsis, and septic shock we found no differences in coagulation parameters analyzed with rotational thromboelastometry between cTnI-positive and -negative patients. According to the results of the present study, other mechanisms than thrombus-associated myocardial damage might play a major role in cTnI-positive patients with severe sepsis and septic shock. Clinical and experimental studies suggest that cytokines, especially TNFα, IL-1β and IL-6 appear to play a pivotal role in mediating the hemodynamic effects and the release of cardiac troponin in patients with severe sepsis and septic shock. Proposed mechanisms are cytokine-mediated reversible myocardial membrane leakage and/or apoptosis.
